# Associations of waist circumference to height ratio and body mass index through childhood and adolescence on blood pressure and risk of young adult hepatic steatosis: a cohort study

**DOI:** 10.1136/archdischild-2024-328140

**Published:** 2025-05-26

**Authors:** Sumona Mandal, Sam D Leary, Nic Timpson, Kushala Abeysekera, Julian P H Shield

**Affiliations:** 1 Population Health Sciences, University of Bristol, Bristol, UK; 2 Bristol Dental School, University of Bristol, Bristol, UK; 3 MRC Integrative Epidemiology Unit, University of Bristol, Bristol, UK; 4 Department of Liver Medicine, Bristol Royal Infirmary, Bristol, UK; 5 NIHR Bristol Biomedical Research Centre, University of Bristol, Bristol, UK; 6 University Hospitals Bristol and Weston NHS Foundation Trust, Bristol, UK

**Keywords:** Child Health, Paediatrics

## Abstract

**Objective:**

Body mass index (BMI), and its adjustment for age and sex as a z-score, is a common measure of excess weight but performs poorly in children. Waist circumference to height ratio (WHtR), which reflects excess adiposity, may be a useful alternative. We explored the associations between WHtR and BMI from childhood through adulthood and hepatic steatosis and systolic blood pressure (SBP).

**Design:**

Participants (n=2193) from the Avon Longitudinal Study of Parents and Children were categorised into eight groups based on criteria for assessing excess adiposity at 7, 15 and 24 years using WHtR≥0.5, and excess weight using established age-specific BMI cut-off values. Regression models were fitted assessing associations between the groups and hepatic steatosis and SBP at age 24.

**Results:**

Adult excess adiposity (WHtR) and excess weight (BMI) both associate with increased risk of hepatic steatosis: risk ratios (RR) 9.3 (95% CI 6.4 to 13.6) and 8.8 (95% CI 5.8 to 13.5), respectively, compared with always having healthy parameters. Excess adiposity in childhood and adulthood increases the associated risk (RR 11.4 (95% CI 6.2 to 20.9)), but not for excess weight, compared with adulthood alone (RR 7.6 (95% CI 3.9 to 15.1)). Both excess adiposity (RR 14.8 (95% CI 10.0 to 21.7)) and weight (RR 11.6 (95% CI 7.1 to 19.0)) in adolescence and adulthood associate with greater risk than adulthood alone. Positive associations with SBP were observed for excess adiposity or weight in adulthood only, childhood and adulthood, and adolescence and adulthood, and for persistent adiposity/weight, using both measures.

**Implications:**

Excess adiposity or weight in early life, continuing to adulthood, elevates hepatic steatosis risk and associates with increased SBP in adulthood. These findings highlight the impact of youth adiposity and weight on later health outcomes. The strong association of WHtR and hepatic steatosis risk suggests it may be an alternative to BMI, with potential use in paediatric risk stratification.

**WHAT IS ALREADY KNOWN ON THIS TOPIC**

- Systematic reviews suggest waist circumference to height ratio (WHtR) outperforms body mass index (BMI) in predicting adult cardiometabolic risk, with fewer studies across early life.

**WHAT THIS STUDY ADDS**

- Our study demonstrates WHtR from childhood to adulthood is comparable to BMI when assessing hepatic steatosis risk and blood pressure. We provide evidence for the significant impact of excess adiposity and weight in childhood and adolescence in addition to adulthood on hepatic steatosis risk. Excess adiposity and weight in childhood and adolescence, in addition to adulthood, are also associated with higher blood pressure in early adulthood.

**HOW THIS STUDY MIGHT AFFECT RESEARCH, PRACTICE OR POLICY**

- Incorporating WHtR in assessments could provide a simple tool to identify individuals at risk of hepatic steatosis and elevated blood pressure, especially in resource-poor settings.

## Introduction

Body mass index (BMI) is the most common measure to assess excess weight and associated cardiometabolic disease, among other poor health outcomes; however, it has limitations. It does not reflect fat distribution and performs poorly in childhood.^1^ BMI cut-offs vary according to age, sex and ethnicity, making public health messaging challenging.

Waist circumference to height ratio (WHtR) is a simple measurement indicating excess adiposity, better accounting for central adiposity than BMI. Furthermore, a ≥0.5 cut-off indicates increased cardiometabolic risk in adults, children and adolescents in Europe and the USA, while ≥0.46 is better for those in other regions.^2 3^ Agbaje used receiver operating characteristic curve analyses to demonstrate a WHtR cut-off of 0.50 for males and 0.52 for females corresponded to the 75th percentile of total fat mass, with an area under the curve of 0.86 (0.83 for females), sensitivity of 0.51 (0.38 for females) and specificity of 0.95.^4^ The National Institute for Health and Care Excellence (NICE) guidelines suggest using WHtR alongside BMI for a more comprehensive health assessment.^5^

Given the impact of poor cardiometabolic health worldwide and adiposity being a major risk factor, understanding the performance of adiposity measurement indices is critical. Metabolic dysfunction-associated steatotic liver disease (MASLD) and hypertension are known risk factors for cardiovascular disease.^6^ Studies have explored WHtR as an adiposity categorisation tool and cardiometabolic health predictor.^7 8^ In a meta-analysis of 31 papers examining adult obesity-related cardiometabolic risk, WHtR had greater discriminatory power compared with BMI and waist circumference (WC).^9^

Early detection and intervention in childhood obesity is important to reduce future cardiometabolic risk. Recently, Agbaje has demonstrated that WHtR is a better adiposity surrogate than BMI in predicting fat mass and discriminating lean mass from childhood to young adulthood.^4^ A meta-analysis by Tao *et al* showed no substantive differences among BMI, WC and WHtR in identifying hypertension or predicting paediatric hypertension risk.^10^ By contrast, other work in children with obesity found that among BMI, weight and WHtR, only WHtR was an independent predictor for MASLD.^11^ Further work shows strong associations between WHtR and MASLD, with a cut-off value of 0.469 indicating high suspicion of MASLD in children and adolescents.^12^ Park *et al* demonstrated that obesity in adulthood plus overweight/obesity in childhood or adolescence increased type 2 diabetes and coronary heart disease risk more than excess adiposity in adulthood alone.^13^ They observed no association with hypertension and did not compare BMI to WHtR. A prospective cohort study showed WHtR ≥0.5 in childhood increased cardiometabolic risk including hypertension in adolescence, with similar associations when defining overweight by BMI; without extending the study to adulthood.^14^

Little research compares WHtR’s performance to other adiposity indices to predict obesity-related health outcomes, specifically hepatic steatosis and hypertension, from childhood to adulthood. This study explores the associations between increased adiposity (measured by WHtR) and weight (measured by BMI) in childhood and adolescence with hepatic steatosis risk and systolic blood pressure (SBP) in adulthood, assessing whether WHtR is a viable alternative to BMI.

## Methods

### Study cohort

This study used population-based longitudinal data from the Avon Longitudinal Study of Parents and Children (ALSPAC) which includes data for 14 541 pregnant women in Avon, UK, who had expected delivery dates between 1 April 1991 and 31 December 1992^15–17^; further details are available at https://www.bristol.ac.uk/alspac/. Of the initial pregnancies, 13 988 children were alive at 1 year. Following further enrolment, the total sample size after age 7 was 15 447 pregnancies resulting in 14 901 children. The ALSPAC study website contains details of all available data through a fully searchable data dictionary and variable search tool (www.bristol.ac.uk/alspac/researchers/our-data/). Informed consent for the use of data collected via questionnaires and clinics was obtained from participants following the recommendations of the ALSPAC Ethics and Law Committee at the time. This study used data from three approximate age points: childhood (7 years), adolescence (15 years) and adulthood (24 years). Participants were excluded if they were pregnant, had active implants (eg, a pacemaker) or had ascites.

### Exposures

Height, weight and WC were collected at approximately 7, 15 and 24 years in clinics. BMI was calculated as weight (kg)/height squared (m^2^). WHtR was determined by dividing WC (cm) by height (cm). Data from participants at 22 years onwards were collected and managed using Research Electronic Data Capture (University of Bristol).^18^ Defining excess weight by BMI at 7 and 15 years was based on the proposed international age-specific BMI cut-off values, with participants categorised as having excess weight if they exceeded BMI cut-offs for overweight.^19^ At 24 years, excess weight was defined by BMI≥25 kg/m^2^.^20^ Excess adiposity according to WHtR was defined as ≥0.5 at all ages.^7^

After classifying participants as either having excess adiposity or not based on their WHtR at the three age points, they were divided into one of eight distinct exposure groups indicating whether and when they had excess adiposity. The eight groups included never having excess adiposity, persistently having excess adiposity and all combinations of excess adiposity status in childhood, adolescence and adulthood, replicating the exposure variable used by Park *et al*.^13^ This process was repeated for excess weight status according to BMI, with the same eight groups.

### Outcomes

The study outcomes were hepatic steatosis (binary) and average SBP (continuous) at 24 years. Quantifying levels of hepatic steatosis used non-invasive liver ultrasound (FibroScan, Echosens 502 Touch, Echosens, Paris) recording controlled attenuation parameter (CAP).^21^ CAP values ≥275 dB/m defined hepatic steatosis on Fibroscan.^22^

Average SBP was calculated from up to three readings using an Omron M6 monitor on the right arm. All participants self-reporting antihypertensive medications had 10 mm Hg added to their average SBP to correct for differences in underlying and recorded blood pressure.^23^

### Confounding variables

Models were minimally adjusted for sex at birth and age recorded at approximately 24 years old. Analyses were further adjusted for birth weight, gestation, adolescent energy intake, adulthood smoking status, adulthood alcohol consumption and socioeconomic position. Birth weight and gestation were recorded as baseline information on all mothers and children. Adolescent energy intake was the mean daily consumption (kilocalories) derived from a 3-day diet diary at 13.5 years, chosen as it provided the closest available dietary data to adulthood. Smoking and alcohol consumption data were recorded from 24 years’ clinic questionnaires. Socioeconomic position was based on mother’s occupation during pregnancy.

### Statistical analysis

Data were analysed using STATA V.17. Study sample characteristics were described as means with SD or number of participants with percentages, as appropriate. Pearson’s correlation coefficients were calculated to assess correlations between WHtR and BMI at each age point. To test the agreement between WHtR and BMI in categories at each age point, cross-tabulations and kappa statistics were calculated.

To assess associations between the outcome of hepatic steatosis and the eight excess adiposity and weight groups defined by WHtR and BMI, Poisson regression models with robust error variance were fitted to generate risk ratios (RR) with 95% CIs.^24^ Linear regression models were fitted to assess associations between the outcome of average SBP and the eight excess adiposity and weight groups defined by WHtR and BMI, to generate regression coefficients with 95% CIs. For both outcomes, models were minimally adjusted for sex and age at adult clinic (model 1), then repeated adjusting for sex, age at adult clinic visit, birth weight, gestation, adolescent energy intake, adulthood smoking status, adulthood alcohol consumption and socioeconomic position based on mother’s occupation (model 2). Minimally adjusted models were repeated, restricting to participants with complete confounder data, to ensure any differences in associations were due to confounder adjustment as opposed to missing data.

## Results

### Description of study sample

2193 participants had exposure data and at least one study outcome recorded, resulting in 2059 participants for the hepatic steatosis and 2187 for the SBP model 1. For model 2, the sample size was 1538 for hepatic steatosis and 1633 for SBP. Descriptive statistics for all variables are shown in table 1, split by sex.

**Table 1:** Descriptive characteristics and confounding variables of participants at ages 7, 15 and 24 in the sample of participants with exposure data and either blood pressure and/or hepatic steatosis data (n=2193)

	Cohort n (%) or mean (SD) n=2193					
	Age 7		Age 15		Age 24	
	Male (n=851)	Female (n=1342)	Male (n=851)	Female (n=1342)	Male (n=851)	Female (n=1342)
Age (years)	7.5 (0.3)	7.4 (0.3)	15.4 (0.2)	15.4 (0.3)	24.5 (0.8)	24.4 (0.7)
Height (cm)	126 (5.6)	125 (5.6)	174 (7.4)	165 (6.0)	180 (6.7)	166 (6.0)
Weight (kg)	25.8 (4.3)	25.5 (4.5)	63.5 (11.2)	58.0 (9.6)	80.1 (14.8)	67.5 (14.2)
BMI	16.0 (1.8)	16.2 (2.0)	20.7 (3.0)	21.3 (3.2)	24.7 (4.2)	24.4 (4.8)
Waist circumference (cm)	56.8 (4.7)	55.6 (4.9)	76.4 (8.7)	76.0 (8.6)	85.7 (11.3)	77.4 (11.3)
WHtR	0.45 (0.03)	0.44 (0.03)	0.44 (0.05)	0.46 (0.05)	0.48 (0.06)	0.47 (0.07)
Excess weight according to BMI	95 (11.2)	197 (14.7)	133 (15.6)	201 (15.0)	344 (40.0)	449 (33.5)
WHtR≥0.5	57 (6.7)	88 (6.6)	91 (10.7)	265 (19.8)	229 (27.0)	320 (23.9)
	**Male (n=851)**			**Female (n=1342)**		
Smoker in adulthood	261 (31.0)			348 (26.0)		
Alcohol consumption in adulthood						
Never	33 (3.9)			52 (3.9)		
Monthly or less	118 (13.9)			303 (22.6)		
2–4 times a month	308 (36.2)			540 (40.2)		
2–3 times a week	302 (35.5)			370 (27.6)		
4 or more times a week	71 (8.3)			59 (4.4)		
Social class based on mother’s occupation						
I	49 (5.8)			60 (4.5)		
II	285 (33.5)			441 (32.9)		
IIInm	275 (32.3)			430 (32.0)		
IIIm	38 (4.5)			90 (6.7)		
IV	31 (3.6)			71 (5.3)		
V	4 (0.5)			14 (1.0)		
Birth weight (g)	3510 (564)			3371 (502)		
Gestation (weeks)	37.4 (10)			37.5 (9.8)		
Energy intake at 13 years old (kcal)	2169 (510)			1789 (434)		

BMI, body mass index; WHtR, waist circumference to height ratio.

### WHtR and BMI

The percentage of the cohort at 7, 15 and 24 years characterised by WHtR as having excess adiposity was 7% (n=145), 16% (n=356) and 25% (n=549), respectively. For BMI and excess weight, the same figures were 13% (n=292), 15% (n=334) and 36% (n=793). Based on WHtR, 68% never had excess adiposity and in 3% it was persistent. Based on BMI, 59% never had excess weight and in 7% it was persistent. Correlation coefficients between WHtR and BMI at 7, 15 and 24 years old were 0.82 (95% CI 0.81 to 0.83), 0.84 (95% CI 0.82 to 0.85) and 0.91 (95% CI 0.90 to 0.92). The kappa values for WHtR and BMI at 7, 15 and 24 years old were 0.58 (95% CI 0.52 to 0.63), 0.65 (95% CI 0.60 to 0.69) and 0.65 (95% CI 0.62 to 0.68).

### Hepatic steatosis

197 participants had hepatic steatosis (9%). Results from models 1 and 2 are shown in table 2 (WHtR) and table 3 (BMI). With little difference between models, results from model 1 are interpreted as the main results to improve precision. Model 1 restricted to participants with complete confounder data showed little difference to model 1 in the full cohort, suggesting differences in associations were due to confounder adjustment rather than missing data (see the online supplemental material). A general linear trend was seen in hepatic steatosis RR across the eight groups for both WHtR and BMI, with RR increasing across the groups.

10.1136/archdischild-2024-328140.supp1 Supplementary data

**Table 2:** Hepatic steatosis risk ratios (RR) for models 1 and 2* in the eight adiposity exposure groups at 7, 15 and 24 years categorised by waist circumference to height ratio (WHtR)

Adiposity pattern according to WHtR	n	Model 1 Hepatic steatosis RR (95% CI)	n	Model 2 Hepatic steatosis RR (95% CI)
	2059		1538	
Never excess adiposity	1409	1	1073	1
Childhood only	24	1.6 (0.2 to 11.2)	20	2.0 (0.3 to 13.8)
Adolescence only	105	1.7 (0.6 to 4.6)	78	2.4 (0.9 to 6.7)
Childhood and adolescence	19	2.2 (0.3 to 14.5)	15	2.7 (0.4 to 18.0)
Adulthood only	269	9.3 (6.4 to 13.6)	196	9.7 (6.2 to 15.1)
Childhood and adulthood	28	11.4 (6.2 to 20.9)	21	15.1 (8.1 to 28.0)
Adolescence and adulthood	145	14.8 (10.0 to 21.7)	98	16.5 (10.5 to 25.9)
Persistent excess adiposity	60	15.0 (9.6 to 23.2)	37	16.6 (9.7 to 28.2)

*Model 1: minimally adjusted for age at adult clinic attendance and sex. Model 2: fully adjusted for age at adult clinic attendance, sex, birth weight, gestation, smoking status in adulthood, alcohol intake in adulthood, energy intake in adolescence and socioeconomic position.

**Table 3:** Hepatic steatosis risk ratios (RR) for models 1 and 2* in the eight weight exposure groups at 7, 15 and 24 years categorised by body mass index (BMI)

Weight pattern according to BMI†	n	Model 1 Hepatic steatosis RR (95% CI)	n	Model 2 Hepatic steatosis RR (95% CI)
	2059		1538	
Never excess weight	1225	1	942	1
Childhood only	50	1.0 (0.1 to 7.4)	34	1.5 (0.2 to 10.6)
Adolescence only	39	1.2 (0.2 to 8.6)	29	1.6 (0.2 to 12.2)
Childhood and adolescence	14	–	10	–
Adulthood only	412	8.8 (5.8 to 13.5)	296	8.9 (5.4 to 14.7)
Childhood and adulthood	63	7.6 (3.9 to 15.1)	45	9.2 (4.4 to 19.3)
Adolescence and adulthood	113	11.6 (7.1 to 19.0)	82	11.5 (6.4 to 20.6)
Persistent excess weight	143	16.4 (10.6 to 25.4)	100	17.4 (10.4 to 29.0)

*Model 1: minimally adjusted for age at adult clinic attendance and sex. Model 2: fully adjusted for age at adult clinic attendance, sex, birth weight, gestation, smoking status in adulthood, alcohol intake in adulthood, energy intake in adolescence and socioeconomic position.

†No cases of hepatic steatosis were seen within the childhood and adolescence group.

Excess adiposity in the adulthood only group had an RR of 9.3 (95% CI 6.4 to 13.6) compared with the group who never had excess adiposity (table 2). Compared with those who never had excess adiposity, the risk of hepatic steatosis was 11.4 (95% CI 6.2 to 20.9) times higher among those with excess adiposity in childhood and adulthood and 14.8 (95% CI 10 to 21.7) times higher for those with excess adiposity in adolescence and adulthood. Persistent excess adiposity had the highest RR of 15.0 (95% CI 9.6 to 23.2). There was no additional risk for those with excess adiposity only in childhood or adolescence compared with never having excess adiposity.

Similar patterns were seen in the eight BMI group categories (table 3). Excess weight in the adulthood only group was associated with an RR of 8.8 (95% CI 5.8 to 13.5) compared with those never having excess weight. Excess weight in adolescence in addition to adulthood increased RR to 11.6 (95% CI 7.1 to 19.0). Persisting excess weight associated with the highest RR at 16.4 (95% CI 10.6 to 25.4). Excess weight in childhood and adulthood did not associate with greater risk than adulthood alone (RR 7.6 (95% CI 3.9 to 15.1) vs 8.8 (95% CI 5.8 to 13.5)). Having excess weight only in childhood or adolescence carried no more risk than those who never had excess weight. Within the group who had excess weight in childhood and adolescence, none had hepatic steatosis.

### Systolic blood pressure

The mean (SD) SBP at 24 years was 115 mm Hg (11 mm Hg). Regression coefficients from models 1 and 2 are shown in tables 4 and 5. With little difference between models 1 and 2, results from model 1 are interpreted to improve precision. Model 1 restricted to participants with complete confounder data showed little difference to model 1 in the full cohort, suggesting differences in associations were due to confounder adjustment rather than missing data (see the online supplemental material).

**Table 4:** Regression coefficients for models 1 and 2* between systolic blood pressure (SBP) and in the eight adiposity exposure groups at 7, 15 and 24 years categorised by waist circumference to height ratio (WHtR)

Adiposity pattern according to WHtR	n	Model 1 SBP regression coefficients mm Hg (95% CI)	n	Model 2 SBP regression coefficients mm Hg (95% CI)
	2187		1633	
Never excess adiposity	1483	–	1124	–
Childhood only	28	0.5 (−3.0 to 4.0)	25	0.8 (−2.9 to 4.5)
Adolescence only	110	1.4 (−0.4 to 3.3)	82	1.8 (−0.4 to 3.9)
Childhood and adolescence	21	−2.2 (−6.2 to 1.9)	16	−1.2 (−5.9 to 3.4)
Adulthood only	293	4.8 (3.6 to 6.0)	216	4.6 (3.2 to 6.0)
Childhood and adulthood	29	4.7 (1.3 to 8.2)	21	4.5 (0.5 to 8.6)
Adolescence and adulthood	157	5.8 (4.3 to 7.4)	106	6.0 (4.1 to 7.9)
Persistent excess adiposity	66	6.6 (4.3 to 8.9)	43	7.0 (4.1 to 9.8)

*Model 1: minimally adjusted for age at adult clinic attendance and sex. Model 2: fully adjusted for age at adult clinic attendance, sex, birth weight, gestation, smoking status in adulthood, alcohol intake in adulthood, energy intake in adolescence and socioeconomic position.

**Table 5:** Regression coefficients for models 1 and 2* between systolic blood pressure (SBP) and in the eight weight exposure groups at 7, 15 and 24 years categorised by body mass index (BMI)

Weight pattern according to BMI	n	Model 1 SBP regression coefficients mm Hg (95% CI)	P value	n	Model 2 SBP regression coefficients mm Hg (95% CI)	P value
	2187			1633		
Never excess weight	1289	–	–	989	–	–
Childhood only	54	−2.0 (−4.5 to 0.6)	0.1	37	−1.8 (−4.9 to 1.3)	0.3
Adolescence only	39	0.2 (−2.8 to 3.2)	0.9	29	−2.0 (−5.4 to 1.5)	0.3
Childhood and adolescence	17	−0.1 (−4.6 to 4.4)	1.0	13	0.8 (−4.3 to 5.9)	0.8
Adulthood only	447	4.9 (3.9 to 5.9)	<0.001	321	4.7 (3.6 to 5.9)	<0.001
Childhood and adulthood	65	6.1 (3.8 to 8.5)	<0.001	45	6.2 (3.5 to 9.0)	<0.001
Adolescence and adulthood	121	5.9 (4.1 to 7.6)	<0.001	89	5.9 (3.9 to 7.9)	<0.001
Persistent excess weight	155	6.3 (4.7 to 7.8)	<0.001	110	6.8 (4.9 to 8.6)	<0.001

*Model 1: minimally adjusted for age at adult clinic attendance and sex. Model 2: fully adjusted for age at adult clinic attendance, sex, birth weight, gestation, smoking status in adulthood, alcohol intake in adulthood, energy intake in adolescence and socioeconomic position.

There was a positive association between SBP and excess adiposity in the adulthood only group (4.8 mmHg (95% CI 3.6 to 6.0)), childhood and adulthood (4.7 mmHg (95% CI 1.3 to 8.2)), adolescence and adulthood (5.8 mmHg (95% CI 4.3 to 7.4)) and persistent excess adiposity (6.6 mmHg (95% CI 4.3 to 8.9)) (table 4). Likewise, there were positive associations between SBP and excess weight in the adulthood only group (4.9 mmHg (95% CI 3.9 to 5.9)), childhood and adulthood (6.1 mmHg (95% CI 3.8 to 8.5)), adolescence and adulthood (5.9 mmHg (95% CI 4.1 to 7.6)) and persistent excess weight (6.3 mmHg (95% CI 4.7 to 7.8)) (table 5). For both WHtR and BMI, across these four groups, a positive linear trend in regression coefficient was seen, with the exception of the adolescence and adulthood group.

## Discussion

### Findings

The findings from this study suggest that having excess adiposity categorised by WHtR in childhood or adolescence, or high BMI in adolescence, in addition to adulthood, increases hepatic steatosis risk and associates with higher SBP compared with never having excess adiposity or weight. This adds further evidence on the relationship between WHtR and BMI through childhood to early adulthood and SBP, showing strong relationships between SBP and excess adiposity or weight in childhood and adolescence in addition to adulthood. This study is novel in its longitudinal examination of WHtR and BMI from childhood to adulthood. The focus on the association of these indices with hepatic steatosis, an increasingly prevalent but relatively less explored pathology, is novel.

The kappa values between WHtR and BMI at the three age points were between 0.58 and 0.65, with WHtR categorising more participants as having excess adiposity in adolescence compared with BMI categorising those with excess weight. According to Cohen’s kappa interpretation, this correlates with weak to moderate agreement.^25^ This is higher agreement than 0.46–0.47 reported by Veldhuis *et al* in 5-year-olds and similar to 0.68 found in adult male army personnel.^26 27^

The positive correlation between WHtR and BMI became stronger with age. This trend aligns with Faria *et al* who demonstrated increasingly positive correlation coefficients through ages <5, 5–10 and >10 years.^28^ The findings contrast with Graves *et al*, who showed strong positive correlations between WHtR and BMI which did not strengthen between childhood (r=0.80) and adolescence (r=0.78).^14^

Hepatic steatosis RRs increased linearly across the eight exposure groups for WHtR and BMI. Compared with hepatic steatosis risk in adulthood alone, additional excess adiposity or weight in childhood or adolescence determined by WHtR, or in adolescence determined by BMI, associates with increased risk. Existing literature has suggested WHtR to be a predictor of childhood MASLD.^11 12^ Interestingly, this present study found that having excess adiposity or weight in childhood or adolescence only did not increase hepatic steatosis risk. This emphasises the importance of targeted weight loss interventions in early life to mitigate future risk. If young people with excess adiposity or weight successfully address this, avoiding adult excess adiposity or weight, their risk of hepatic steatosis could match those never categorised as such. Yan *et al* also suggested that MASLD risk in adulthood associated with being overweight/obese in childhood, measured by BMI or subscapular skinfold thickness, could be mitigated by attaining adult non-obese status.^29^

This study suggests associations between SBP and excess adiposity and weight in adulthood only, childhood and adulthood, adolescence and adulthood and persistent excess adiposity and weight categorised by WHtR and BMI. Having excess adiposity or weight in childhood, adolescence or both did not show higher regression coefficients compared with never having excess adiposity or weight. This contrasts with previous work suggesting that being overweight in early life did not increase the risk of hypertension in adults with obesity.^13^ This discrepancy may be explained by our cohort’s higher percentage with excess adiposity or weight. This supports other data suggesting that adults with obesity who were overweight/obese as children had increased hypertension risk and other adverse cardiovascular outcomes.^30^ Like this present study, this work suggested that if children with increased adiposity or weight reduced to healthy weight by adulthood, the risk of adverse outcomes was similar to those never obese. GLP-1 agonists, alongside lifestyle interventions in youth, result in greater reductions in BMI than lifestyle measures alone.^31 32^ A systematic review showed large BMI reductions and other weight indices with semaglutide and behavioural interventions with higher contact hours and physical activity sessions.^33^ However, limited follow-up for these interventions requires scrutiny, highlighting the need for longer term studies to evaluate sustained effectiveness.

Excess BMI and WHtR associate with higher hepatic steatosis RR across most exposure groups. Regression coefficients for SBP were similar between measures. WHtR has advantages over BMI as it is more simple and quicker to measure, does not require accurate weight scales, reflects central adiposity and has a single cut-off value rather than needing reference to myriad percentile charts or calculating z-scores, making it a viable alternative to BMI in many less resourced community settings.

### Limitations

The relatively small sample size in the childhood and adolescence exposure group, with no cases of hepatic steatosis categorised by BMI, reduces statistical robustness and should be replicated with a larger sample. The cohort’s lack of ethnic diversity (~96% ethnically white) limits generalisability, especially given certain ethnicities have increased cardiometabolic risk. The lack of complete physical activity data precluded physical activity being adjusted for in the analysis, which is a potential confounder. Additional covariates which putatively may influence outcome beyond the analysed adiposity measures, such as insulin resistance and dyslipidaemia, were found previously in the same cohort to add little predictive value and were not included in this analysis.^34^

The ALSPAC study population reflects an affluent cohort, subject to attrition bias over three decades, with lower obesity prevalence than the general population. After excluding participants with missing outcome and confounder data, our sample size reduced, which may reduce the representativeness of the results. Furthermore, there are statistical power limitations imposed in any time-limited recruitment cohort, with loss of participants and missed data point collections in follow-ups at older ages.

## Conclusion

This study supports NICE guidelines recommending incorporation of WHtR alongside BMI in assessing adiposity and health risks in all age groups. It also demonstrates the increased health risks attendant on excess adiposity and weight in childhood and adolescence compared with adulthood alone. Targeted strategies throughout childhood to reduce adiposity would reduce adult cardiometabolic risk, with WHtR a viable alternative to BMI in risk-stratifying children.

## Data Availability

Data may be obtained from a third party and are not publicly available. Authors using ALSPAC study data cannot share or provide access to data outside of the approved contributors listed on this paper. ALSPAC is only able to share data that have been requested, justified and approved via a submitted proposal or amendment. The online form for this process can be found on the ALSPAC website. The ALSPAC study website contains details of all the data available through a fully searchable data dictionary and variable search tool: http://www.bristol.ac.uk/alspac/researchers/our-data/
