# Supplementary material for: Associations of waist circumference to height ratio and body mass index through childhood and adolescence on blood pressure and risk of young adult hepatic steatosis: a cohort study
Source: Arch Dis Child. 2025 May 26;110(11):885–91. doi: 10.1136/archdischild-2024-328140 (PMC12573424; doi:10.1136/archdischild-2024-328140)
Supplement: Supplementary data [file archdischild-110-11-s001.pdf]

Supplementary material

Table S1: Hepatic steatosis risk ratios (RR) in the eight adiposity exposure groups at 7, 15 and 24 years categorised by waist circumference to height ratio (WHtR)

**Adiposity pattern according to WHtR**	**N**	**Minimally adjusted* hepatic steatosis RR (95% CI)**	**N**	**Minimally adjusted* hepatic steatosis RR in restricted** sample size (95% CI)**	**N**	**Fully adjusted** hepatic steatosis RR (95% CI)**
	**2059**		**1538**		**1538**	
Never excess adiposity	1409	1	1073	-	1073	1
Childhood only	24	1.6 (0.2, 11.2)	20	2.1 (0.3 , 14.3)	20	2.0 (0.3, 13.8)
Adolescence only	105	1.7 (0.6, 4.6)	78	2.4 (0.9 , 6.7)	78	2.4 (0.9, 6.7)
Childhood and adolescence	19	2.2 (0.3, 14.5)	15	2.9 (0.4, 19.5)	15	2.7 (0.4, 18.0)
Adulthood only	269	9.3 (6.4, 13.6)	196	10.0 (6.4 , 15.6)	196	9.7 (6.2, 15.1)
Childhood and adulthood	28	11.4 (6.2, 20.9)	21	15.6 (8.4, 28.0)	21	15.1 (8.1, 28.0)
Adolescence and adulthood	145	14.8 (10.0, 21.7)	98	17.9 (11.4 , 28.0)	98	16.5 (10.5, 25.9)
Persistent excess adiposity	60	15.0 (9.6, 23.2)	37	18.0 (10.6, 30.5)	37	16.6 (9.7, 28.2)

CI, confidence intervals
*Sample size minimally adjusted for confounders age at adult clinic attendance and sex
**Sample size fully adjusted for confounders age at adult clinic attendance, sex, birthweight, gestation, smoking status in adulthood, alcohol intake in adulthood, energy intake in adolescence, social class

Table S2: Hepatic steatosis risk ratios (RR) in the eight weight exposure groups at 7, 15 and 24 years categorised by body mass index (BMI)

**Weight pattern according to BMI*** **	**N**	**Minimally adjusted* hepatic steatosis RR (95% CI)**	**N**	**Minimally adjusted* hepatic steatosis RR in restricted** sample size (95% CI)**	**N**	**Fully adjusted** hepatic steatosis RR (95% CI)**
	**2059**		**1538**		**1538**	
Never excess weight	1225	1	942	1	942	1
Childhood only	50	1.0 (0.1, 7.4)	34	1.6 (0.2 , 11.5)	34	1.5 (0.2, 10.6)
Adolescence only	39	1.2 (0.2, 8.6)	29	1.7 (0.2 , 12.1)	29	1.6 (0.2, 12.2)
Childhood and adolescence	14	-	10	-	10	-
Adulthood only	412	8.8 (5.8, 13.5)	296	9.2 (5.6 , 15.1)	296	8.9 (5.4, 14.7)
Childhood and adulthood	63	7.6 (3.9, 15.1)	45	10.0 (4.8 , 20.8)	45	9.2 (4.4, 19.3)
Adolescence and adulthood	113	11.6 (7.1, 19.0)	82	12.4 (7.0 , 22.0)	82	11.5 (6.4, 20.6)
Persistent excess weight	143	16.4 (10.6, 25.4)	100	18.7 (11.3, 31.0)	100	17.4 (10.4, 29.0)

CI, confidence intervals

*Sample size minimally adjusted for confounders age at adult clinic attendance and sex

**Sample size fully adjusted for confounders age at adult clinic attendance, sex, birthweight, gestation, smoking status in adulthood, alcohol intake in adulthood, energy intake in adolescence, social class

***No cases of hepatic steatosis were seen within the childhood and adolescence group.

Table S3: Regression coefficients between systolic blood pressure (SBP) and the eight adiposity exposure groups at 7, 15 and 24 years categorised by waist circumference to height ratio (WHtR).

**Adiposity pattern according to WHtR**	**N**	**Minimally adjusted* SBP regression coefficient, mmHg (95% CI)**	**N**	**Minimally adjusted* SBP regression coefficient in restricted** sample size, mmHg (95% CI)**	**N**	**Fully adjusted** SBP regression coefficient , mmHg (95% CI)**
	**2187**		**1633**		**1633**	
Never excess adiposity	1483	-	1124	-	1124	-
Childhood only	28	0.5 (-3.0,4.0)	25	0.4 (-3.4, 4.1)	25	0.8 (-2.9, 4.5)
Adolescence only	110	1.4 (-0.4, 3.3)	82	1.5 (-0.6, 3.6)	82	1.8 (-0.4, 3.9)
Childhood and adolescence	21	-2.2 (-6.2, 1.9)	16	-1.9 (-6.6, 2.7)	16	-1.2 (-5.9, 3.4)
Adulthood only	293	4.8 (3.6, 6.0)	216	4.5 (3.1, 5.9)	216	4.6 (3.2, 6.0)
Childhood and adulthood	29	4.7 (1.3, 8.2)	21	4.2 (0.1, 8.2)	21	4.5 (0.5, 8.6)
Adolescence and adulthood	157	5.8 (4.3, 7.4)	106	5.6 (3.7, 7.5)	106	6.0 (4.1, 7.9)
Persistent excess adiposity	66	6.6 (4.3, 8.9)	43	6.4 (3.5, 9.2)	43	7.0 (4.1, 9.8)

CI, confidence intervals

*Sample size minimally adjusted for confounders age at adult clinic attendance and sex

**Sample size fully adjusted for confounders age at adult clinic attendance, sex, birthweight, gestation, smoking status in adulthood, alcohol intake in adulthood, energy intake in adolescence, social class

Table S4: Regression coefficients between systolic blood pressure (SBP) and the eight weight exposure groups at 7, 15 and 24 years categorised by body mass index (BMI).

**Weight pattern according to BMI**	**N**	**Minimally adjusted* SBP coefficient, mmHg (95% CI)**	**N**	**Minimally adjusted* SBP regression coefficient in restricted** sample size, mmHg (95% CI)**	**N**	**Fully adjusted** SBP regression coefficient, mmHg (95% CI)**
	**2187**		**1633**		**1633**	
Never excess weight	1289	-	989	-	989	-
Childhood only	54	-2.0 (-4.5 , 0.6)	37	-2.2 (-5.3, 0.8)	37	-1.8 (-4.9, 1.3)
Adolescence only	39	0.2 (-2.8,3.2)	29	-2.3 (-5.7, 1.1)	29	-2.0 (-5.4,1.5)
Childhood and adolescence	17	-0.1 (-4.6, 4.4)	13	-0.1 (-5.2, 5.0)	13	0.8 (-4.3, 5.9)
Adulthood only	447	4.9 (3.9 , 5.9)	321	4.6 (3.4, 5.8)	321	4.7 (3.6, 5.9)
Childhood and adulthood	65	6.1 (3.8 , 8.5)	45	5.8 (3.0, 8.6)	45	6.2 (3.5, 9.0)
Adolescence and adulthood	121	5.9 (4.1, 7.6)	89	5.4 (3.4, 7.4)	89	5.9 (3.9, 7.9)
Persistent excess weight	155	6.3 (4.7, 7.8)	110	6.1 (4.3, 8.0)	110	6.8 (4.9, 8.6)

CI, confidence intervals

*Sample size minimally adjusted for confounders age at adult clinic attendance and sex

**Sample size fully adjusted for confounders age at adult clinic attendance, sex, birthweight, gestation, smoking status in adulthood, alcohol intake in adulthood, energy intake in adolescence, social class
